# Model predictions of global geologic hydrogen resources

**DOI:** 10.1126/sciadv.ado0955

**Published:** 2024-12-13

**Authors:** Geoffrey S. Ellis, Sarah E. Gelman

**Affiliations:** Energy Resources Program, U.S. Geological Survey, Denver, CO, USA.

## Abstract

Geologic hydrogen could be a low-carbon primary energy resource; however, the magnitude of Earth’s subsurface endowment has not yet been assessed. Knowledge of the occurrence and behavior of natural hydrogen on Earth has been combined with information from geologic analogs to construct a mass balance model to predict the resource potential. Given the associated uncertainty, stochastic model results predict a wide range of values for the potential in-place hydrogen resource [10^3^ to 10^10^ million metric tons (Mt)] with the most probable value of ~5.6 × 10^6^ Mt. Although most of this hydrogen is likely to be impractical to recover, a small fraction (e.g., 1 × 10^5^ Mt) would supply the projected hydrogen needed to reach net-zero carbon emissions for ~200 years. This amount of hydrogen contains more energy (~1.4 × 10^16^ MJ) than all proven natural gas reserves on Earth (~8.4 × 10^15^ MJ). Study results demonstrate that further research into understanding the potential for geologic hydrogen resources is merited.

## Teaser

A model of global geologic hydrogen resources predicts a substantial low-carbon energy resource potential in the subsurface.

## INTRODUCTION

Hydrogen is projected to account for as much as 30% of the future energy supply in some sectors, with the global demand increasing more than fivefold by 2050 (*1*). To achieve net-zero carbon goals, the future supply of hydrogen is expected to be obtained from the electrolysis of water using renewable electricity (also known as green hydrogen) and from fossil fuel sources coupled with carbon capture, utilization, and storage (also known as blue hydrogen) (*2*). However, realization of these production levels will require development of infrastructure at an unprecedented rate (*3*), as well as substantial contributions from technologies that are not commercially viable today (*2*). In addition, hydrogen production may not be as climate friendly as previously assumed (*4*–*6*). Now, hydrogen is generally viewed as a medium for energy storage and transport and not a primary resource (*7*). However, a recent discovery of a substantial accumulation of natural hydrogen in Mali, Africa (*8*–*10*) has challenged the long-held view that such fields do not exist (*11*, *12*). There is a growing recognition among geoscientists that suitable exploration tools have not been deployed in the appropriate locations to truly evaluate the resource potential of natural hydrogen in the Earth’s subsurface (*11*–*15*). Information regarding the resource potential of geologic hydrogen can support policy-makers, resource managers, exploration companies, and investors in the decision-making process. However, the uncertainties associated with the generation, migration, accumulation, and preservation of hydrogen in the subsurface make it impossible to precisely determine potential volumes at this time.

**Fig. 1.**
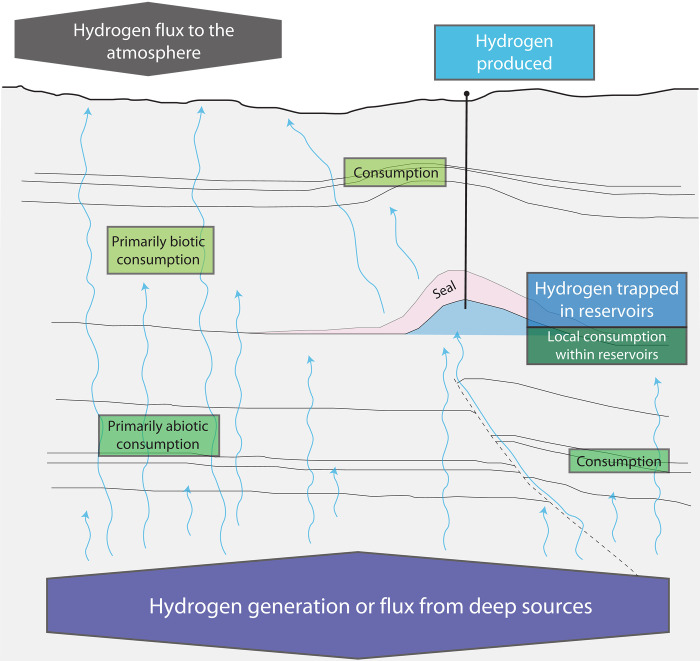
Conceptual model of geologic hydrogen resources. The model inputs include annual generation of natural hydrogen, fraction of hydrogen detained in traps, residence time in reservoirs, proportion of biotic and abiotic loss, and the rate of anthropogenic production. The calculated outputs of the model are the amount of hydrogen stored in reservoirs at a given time and the flux to the atmosphere.

A recent compilation of published studies on the global generation of natural hydrogen in all geologic settings estimates the amount to be 15 to 31 million metric tons (Mt or 10^9^ g) per year (*16*). Because the global demand for hydrogen is projected to reach ~530 Mt year^−1^ by the year 2050 (*1*), production of all the annually generated hydrogen in the Earth’s subsurface would likely represent a small fraction of the needed supply. However, the resource potential for geologic hydrogen is not only dependent on the generation rate but also on the propensity for hydrogen to become trapped in the subsurface and for accumulations to be preserved. Although there is uncertainty related to the presence of hydrogen in the subsurface, much is known about its occurrence and behavior (*16*). Additional inferences can be gained by using knowledge derived from studies of fluid migration, accumulation, and preservation in other fields such as petroleum geology, geothermal energy, and hydrothermal minerals. Information from these studies can be combined to provide some constraints on the possible magnitude of geologic hydrogen resources in the subsurface. We present here a model of the global potential geologic hydrogen resource based on a mass balance approach (Fig. 1). The model results are compared with the projected demand for hydrogen to determine whether natural hydrogen might meet a sufficient portion of the future demand to merit further investigation and exploration. The results can also provide some insight into the most impactful factors affecting the geologic hydrogen resource potential, highlighting the areas that could be a priority for research efforts.

## RESULTS

**Fig. 2.**
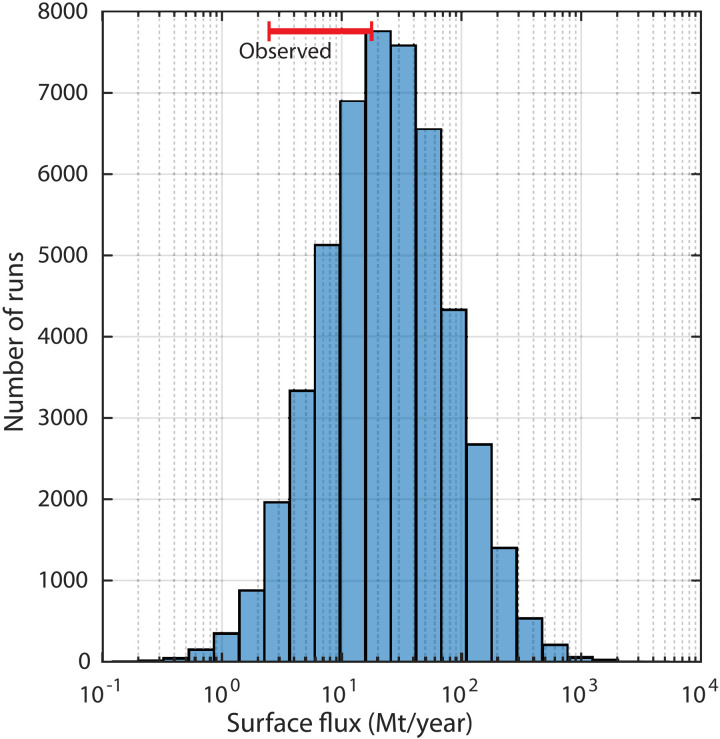
Estimated annual hydrogen flux to the atmosphere. The sum of the amount of generated hydrogen not trapped or consumed plus the amount that leaks out of reservoirs and is not consumed is considered flux to the atmosphere (blue bars). Volcanic and hydrothermal settings are thought to be the single largest source of hydrogen from the subsurface, contributing ~9.6 ± 7.2 Mt year^−1^ to the atmosphere (red bar) (*17*). Additional contributions of hydrogen to the atmosphere from terrestrial macro- and microseeps are not well constrained (*16*) and could account for the additional predicted flux.

The calculated annual flux of hydrogen from the subsurface to the atmosphere, which is the sum of the nontrapped and leaked hydrogen less the amount consumed by biotic and abiotic processes, was compared to published estimates as a check on our model calculations (Fig. 2). The estimated natural flux to the atmosphere from our model ranges from <1 to ~1 × 10^3^ Mt year^−1^ with the most probable value of ~24 Mt year^−1^ (mean of ~50 Mt year^−1^). The largest known fluxes of natural hydrogen from the subsurface to the atmosphere are thought to be from volcanic and hydrothermal settings, which are estimated to be ~9.6 ± 7.2 Mt year^−1^ (*17*). The high end of this estimate (16.8 Mt year^−1^) is similar to the most probable estimated natural flux of hydrogen from the subsurface predicted by our model. Additional contributions of hydrogen to the atmosphere from terrestrial macro- and microseeps are not well constrained and could easily account for a substantial portion of the estimated flux (*16*–*19*). Most (~75%) of the natural flux of hydrogen to the atmosphere is thought to be taken up by soils, but there are large uncertainties associated with the magnitude and mechanisms (*20*). Soils could be consuming a substantially larger amount of hydrogen than currently estimated or other hydrogen sinks on the Earth’s surface may yet be recognized.

**Fig. 3.**
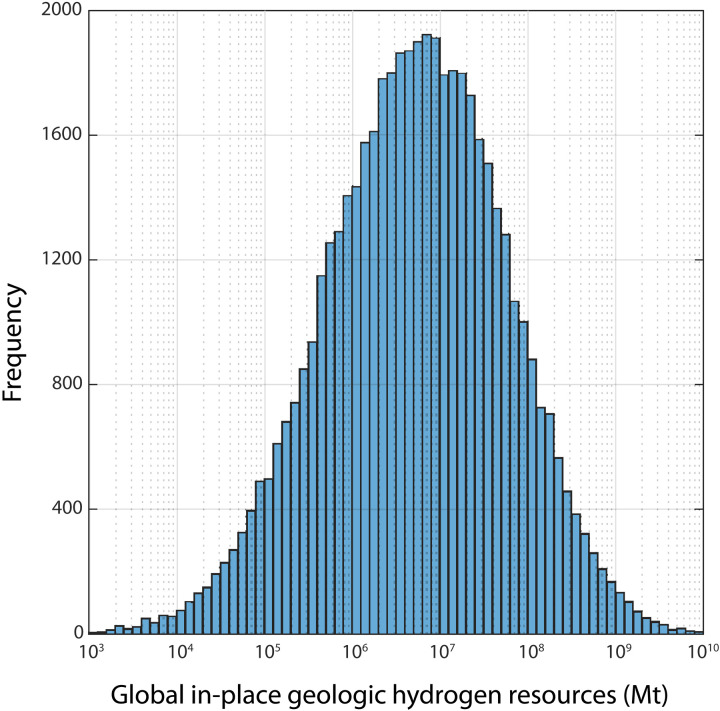
Distribution of predicted amounts of in-place geologic hydrogen resources. Values range from 10^3^ to 10^10^ Mt, with ~5.6 × 10^6^ Mt being the most likely value (P50) and a mean value of ~6.8 × 10^7^ Mt.

The magnitude of the global in-place geologic hydrogen resource today, before anthropogenic production, can be calculated from the mass balance model equations. The calculated total global amount of natural hydrogen in the subsurface ranges from 10^3^ to 10^10^ Mt of hydrogen, with the most probable value of ~5.6 × 10^6^ Mt (mean of ~6.8 × 10^7^ Mt) (Fig. 3). Calculated correlation coefficients specify the relative contribution of each of the model inputs on the output distribution. These values indicate that the residence time in reservoirs associated with biological consumption has the largest impact on the predicted geologic hydrogen resource potential, followed by the natural generation rate (Table 1). The magnitude of hydrogen consumption associated with migration and the amount of hydrogen leakage from reservoirs have negligible effects on the predicted resource potential.

**Table 1.** Model input values and output correlation coefficients. Minimum, maximum, and midpoint values for the input values summarized from literature sources and used in the model calculations. Ranges of input values were normally distributed as shown in fig. S2. Correlation coefficients were calculated with the model outputs.

Input parameter	Min	Mid	Max	Correlation coefficient
H_2_ generation (Mt year^−1^)	25	500	25 × 10^3^	0.44
Residence time due to trap leaking (years)	1 × 10^5^	5 × 10^7^	5 × 10^9^	0.09
Residence time due to consumption in reservoir (years)	1 × 10^4^	1.4 × 10^6^	5 × 10^9^	0.72
Trapping efficiency (fraction)	0.001	0.01	0.1	0.30
Consumption (fraction)	0.9	0.95	0.99999	0.0014
Shallow proportion (fraction)	0.9	0.99	0.999	0.016
Deep proportion (fraction)	0.1	0.01	0.001	−0.016

## DISCUSSION

Given the uncertainties in the model construction and the inputs, the model results should be viewed as a first-order approximation of the magnitude of the potential in-place geologic hydrogen resource. The model makes no predictions about the distribution of the hydrogen in the subsurface, which is critical for the economic viability of any potential resource (*21*). Given what is known about the distribution of petroleum and nonpetroleum fluids (e.g., helium and CO_2_) in the subsurface, it is likely that recovery of most subsurface hydrogen can be expected to be in accumulations that are too deep, too far offshore, or too small to be economically recovered. However, if even a small amount of the most probable predicted in-place resource (~5.6 × 10^6^ Mt) was recoverable, this could represent a substantial resource. The global demand for hydrogen is projected to reach ~500 Mt year^−1^ by 2050 (*1*), and recovery of just 2% of the estimated most probable in-place resource would meet the entire projected global hydrogen demand for ~200 years. Moreover, we calculate the energy content of this estimated recoverable amount of hydrogen (~1 × 10^5^ Mt) to be ~1.4 × 10^16^ MJ, which is roughly twice the amount of energy in all the proven natural gas reserves on Earth (~8.4 × 10^15^ MJ).

Our in-place resource estimate is only for natural hydrogen potentially stored in accumulations in the subsurface. It has been suggested that the rate of hydrogen generation may be sufficiently fast such that it could be economically produced from subsurface fluxes without the need for a reservoir, trap, and seal (*14*, *22*). Additionally, it is possible that natural hydrogen production could be stimulated to increase the rate of generation or induce generation in settings where it has the potential but is not naturally doing so (*23*). Although the magnitude of the potential contributions of hydrogen from natural and stimulated generation in real time is currently unconstrained, these contributions could constitute substantial additions to the in-place resource thought to exist in subsurface reservoirs.

**Fig. 4.**
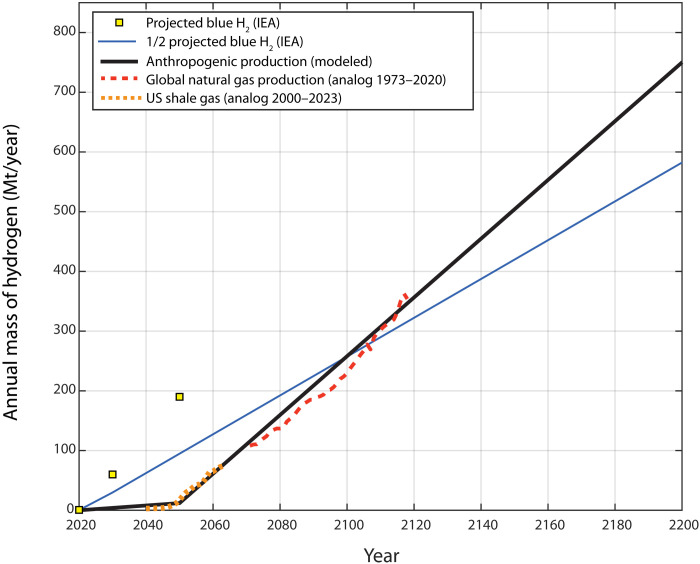
Model and analog trends utilized for modeling future anthropogenic production of hydrogen. The bold black curve corresponds to the modeled annual production of H_2_ implemented in this study. For comparison, the solid blue curve illustrates a continuous trend for half of projected blue hydrogen production based on the International Energy Agency (IEA) estimates (yellow squares) (*1*). Analogs from historical natural gas production are converted from produced natural gas volumes to mass of hydrogen, for both US shale gas (*24*) and global natural gas (*71*).

Of equal importance to the magnitude of the potential resource is the time that may be required to develop it. A ready supply of low-carbon hydrogen will only make a meaningful contribution toward meeting net-zero carbon emission goals if it can be developed in years or decades rather than centuries (*1*). While the development of petroleum resources has taken over a century to reach maturity, there is a good reason to believe that natural hydrogen resources can be developed much more quickly. Although not a perfect analog, the experience of US shale gas resource development suggests that geologic hydrogen could begin to make a substantial contribution to the global energy supply within decades (*24*). Our model predicts that geologic hydrogen production rates could provide half of the projected supply of blue hydrogen by the end of this century, which would substantially reduce the necessary capacity for carbon capture, utilization, and storage (Fig. 4). The rate of progress toward realizing potential geologic hydrogen resources will depend, in large part, on the level of investment in the development of exploration and production strategies and associated technologies. Furthermore, there is a ~94% probability that the subsurface endowment of natural hydrogen will exceed future extraction capacity through the year 2100 and a >75% probability of this being the case beyond the year 2200.

**Fig. 5.**
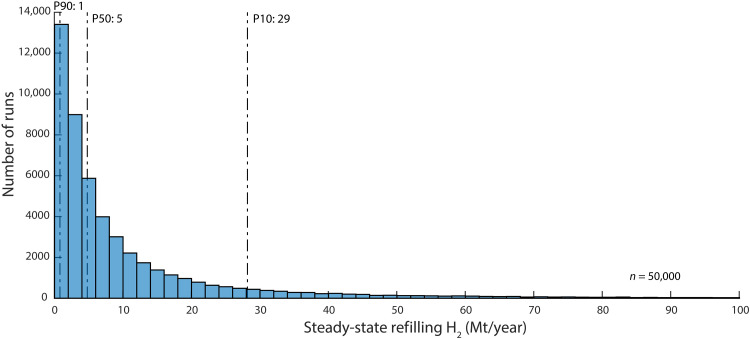
Predicted renewable geologic hydrogen potential. Results of the steady-state refilling (newly generated, migrated, and trapped) hydrogen termed “renewable” hydrogen.

Several recent studies have claimed that natural hydrogen generation rates are rapid enough to potentially offset anthropogenic extraction rates from reservoirs, thereby constituting a renewable resource (*8*, *9*, *14*). Using our hydrogen production model based on historical natural gas production, the most probable (P50) global renewable hydrogen production rate is estimated to be about 5 Mt year^−1^ (P90 = 1 Mt; P10 = 29 Mt) (Fig. 5), which would meet <1% of the projected worldwide demand for hydrogen in 2050 (*1*). Slower hydrogen extraction rates could increase the amount of renewable resource produced annually but would reduce the contribution that natural hydrogen would have toward decarbonizing the energy supply. However, our model does not account for potential geologic hydrogen that might be produced as it is generated or moves through the subsurface, which would be a renewable resource. This form of geologic hydrogen production is totally hypothetical, and the magnitude of this resource cannot currently be estimated.

Our model provides an initial framework for assessment of the global resource potential of natural hydrogen. The estimated amount of in-place hydrogen in the Earth’s subsurface is highly uncertain, varying over seven orders of magnitude; however, the predicted flux to the atmosphere is less variable (three orders of magnitude), with the most probable value roughly within a factor of 2 of current observations. The approach can be improved as more knowledge is acquired and would benefit from geographic and stratigraphic constraints. The study results indicate that a substantial hydrogen resource could exist in the subsurface of Earth, the magnitude of which, if proven, could substantially contribute to the decarbonization of energy resources but is not likely to be renewable. These findings indicate that further research in this field is warranted. A better understanding of the rates and controls on geologic hydrogen consumption in subsurface accumulations and more accurate estimates of the rates of natural hydrogen generation would improve model predictions of the resource potential. Realization of potential natural hydrogen resources will require a more advanced understanding of the processes that lead to the accumulation of hydrogen in the subsurface as well as optimized methods for finding these resources.

## MATERIALS AND METHODS

### Derivation of mass balance model equation

To constrain the estimated subsurface resource potential of geologic hydrogen, we have taken a simple mass balance approach describing the expected sources and sinks of naturally occurring hydrogen in the Earth’s crust (fig. S1). The flux of geologic hydrogen generation in the deep subsurface is considered the main source of hydrogen to the model. The main geologic sink is biotic or abiotic consumption. Hydrogen that is generated geologically either can be trapped in a subsurface reservoir or is never trapped and leaks directly to the surface. In the former case, we consider that hydrogen may be consumed in the reservoir and that traps may leak over geologic timescales. Here, we derive the mass balance equations of the model as shown schematically in the Supplementary Materials (fig. S1); a summary of nomenclature is provided in table S1.

We define the geologic generative flux of hydrogen in the subsurface as $\frac{\partial M_{P}}{\partial t}$, which is equal to the surface flux of hydrogen, $\frac{\partial M_{S}}{\partial t}$, plus the flux of hydrogen that is consumed either biologically or abiotically in the subsurface, $\frac{\partial M_{B}}{\partial t}$

$$\frac{\partial M_{P}}{\partial t}=\frac{\partial M_{S}}{\partial t}+\frac{\partial M_{B}}{\partial t}$$ (1)

This surface flux is composed of hydrogen that was generated and either never trapped in a subsurface reservoir (“never trapped”) or trapped and subsequently leaked over geologic time (“leaked”). Hydrogen that was trapped but never leaks would not migrate to the surface and thus would not be included in the surface flux. Biologic or abiotic consumption reduces the amount of hydrogen that was generated along both routes to the surface. To characterize the proportion of hydrogen that is generated and trapped, we define the trapping efficiency, ϵ.

The portion of the surface flux that was never trapped (and remains after biotic/abiotic consumption) is

$${\left(\frac{\partial M_{S}}{\partial t}\right)}_{\text{NT}}=\left(1-\epsilon \right)\left(1-f_{B}\right)\frac{\partial M_{P}}{\partial t}$$ (2)

The portion of the surface flux that was trapped but leaked requires a rate at which hydrogen leaks from reservoirs in the subsurface, denoted $\frac{\partial M_{L}}{\partial t}$. For simplicity, we consider this as a time-dependent decay process, wherein a decay constant, λ, describes the half-life of trapped hydrogen, the residence time is denoted τ, and the mass of the hydrogen trapped in reservoirs at any moment in time is $M_{R}$

$$\frac{\partial M_{L}}{\partial t}=\lambda_{L}M_{R}=\frac{M_{R}}{\tau_{L}}$$ (3)

Next, the rates of biotic and abiotic consumption must be defined. We consider four terms to capture these processes: (i) consumption that occurs during migration, focused on hydrogen that is never trapped in reservoirs; (ii) consumption that occurs during migration, focused on hydrogen migrating in the deep subsurface at high temperature and likely before trapping; (iii) consumption that occurs during migration, focused on hydrogen migrating in the shallow subsurface at low temperature and likely occurring to hydrogen after it has leaked from traps; and (iv) consumption that occurs locally within reservoirs. Further explanation on the efficiency of deep (likely dominated by abiotic processes) versus shallow consumption (likely dominated by biotic processes) is provided in the “Biotic and abiotic loss” section.

For simplicity, we consider the consumption of hydrogen occurring during migration to be proportional in magnitude to the overall generative flux of hydrogen, where $f_{B}$ denotes the proportion of hydrogen that is consumed along migration pathways

$$\frac{\partial M_{B}}{\partial t}=f_{B}\frac{\partial M_{P}}{\partial t}$$ (4)

We further assume biotic/abiotic consumption during migration to be more efficient in the shallow subsurface at low temperature; thus, a higher proportion of hydrogen is likely to be consumed after hydrogen has leaked from reservoirs in route to the surface, while a lower proportion may be consumed before hydrogen being trapped in reservoirs. We define a parameter, x, as the proportion of consumption that occurs in the shallow subsurface. Last, the portion of surface flux that was leaked (and remains after biotic/abiotic consumption) is

$${\left(\frac{\partial M_{S}}{\partial t}\right)}_{L}={\left(1-f_{B}\right)}^{x}\frac{\partial M_{L}}{\partial t}={\left(1-f_{B}\right)}^{x}\frac{M_{R}}{\tau_{L}}$$ (5)

The total surface flux of both never trapped and leaked hydrogen is obtained by combining Eqs. 2 and 5

$$\begin{array}{c} \frac{\partial M_{S}}{\partial t}={\left(\frac{\partial M_{S}}{\partial t}\right)}_{\text{NT}}+{\left(\frac{\partial M_{S}}{\partial t}\right)}_{L}=\left(1-\epsilon \right)\left(1-f_{B}\right)\frac{\partial M_{P}}{\partial t}\\ +{\left(1-f_{B}\right)}^{x}\frac{M_{R}}{\tau_{L}} \end{array}$$ (6)

To define the hydrogen loss term in Eq. 1, we must consider the multiple sinks outlined above associated with consumption, following the terms defined in fig. S1. The hydrogen that was never trapped but consumed during migration is

$${\left(\frac{\partial M_{B}}{\partial t}\right)}_{\text{NT}}=\left(1-\epsilon \right)f_{B}\frac{\partial M_{P}}{\partial t}$$ (7)

The hydrogen that is consumed at depth before being trapped in reservoirs is

$${\left(\frac{\partial M_{B}}{\partial t}\right)}_{\text{PTD}}=\epsilon \left[1-{\left(1-f_{B}\right)}^{1-x}\right]\frac{\partial M_{P}}{\partial t}$$ (8)

The hydrogen that is consumed at shallow depths after being leaked from reservoirs is

$${\left(\frac{\partial M_{B}}{\partial t}\right)}_{\text{PTS}}=\left[1-{\left(1-f_{B}\right)}^{x}\right]\frac{M_{R}}{\tau_{L}}$$ (9)

Although stored within reservoirs, we also consider the loss of hydrogen to local biotic consumption. This process is also modeled as a time-dependent decay process. We consider the change in the mass of hydrogen in the reservoir due to local consumption to be

$${\left(\frac{\partial M_{B}}{\partial t}\right)}_{R}=\lambda_{C}M_{R}=\frac{M_{R}}{\tau_{C}}$$ (10)

The total sum of all terms relating to consumption therefore is

$$\frac{\partial M_{B}}{\partial t}={\left(\frac{\partial M_{B}}{\partial t}\right)}_{\text{NT}}+{\left(\frac{\partial M_{B}}{\partial t}\right)}_{\text{PTD}}+{\left(\frac{\partial M_{B}}{\partial t}\right)}_{\text{PTS}}+{\left(\frac{\partial M_{B}}{\partial t}\right)}_{R}$$ (11)

We can relate this total surface flux and the total consumption-associated losses back to the total mass balance, combining Eqs. 1, 6, 11 and simplifying

$$\begin{array}{c} \frac{\partial M_{P}}{\partial t}=\left(1-\epsilon \right)\left(1-f_{B}\right)\frac{\partial M_{P}}{\partial t}+{\left(1-f_{B}\right)}^{x}\frac{M_{R}}{\tau_{L}}+\left(1-\epsilon \right)f_{B}\frac{\partial M_{P}}{\partial t}\\ +\epsilon \left[1-{\left(1-f_{B}\right)}^{1-x}\right]\frac{\partial M_{P}}{\partial t}+\left[1-{\left(1-f_{B}\right)}^{x}\right]\frac{M_{R}}{\tau_{L}}+\frac{M_{R}}{\tau_{C}} \end{array}$$

Expanding parenthesis

$$\begin{array}{c} \frac{\partial M_{P}}{\partial t}=\left[1-f_{B}-\epsilon +\epsilon f_{B}\right]\frac{\partial M_{P}}{\partial t}+{\left(1-f_{B}\right)}^{x}\frac{M_{R}}{\tau_{L}}+\left(f_{B}-\epsilon f_{B}\right)\frac{\partial M_{P}}{\partial t}\\ +\left[\epsilon -\epsilon {\left(1-f_{B}\right)}^{1-x}\right]\frac{\partial M_{P}}{\partial t}+\frac{M_{R}}{\tau_{L}}-{\left(1-f_{B}\right)}^{x}\frac{M_{R}}{\tau_{L}}+\frac{M_{R}}{\tau_{C}} \end{array}$$

Removing cancelled terms

$$\begin{array}{c} \frac{\partial M_{P}}{\partial t}=\frac{\partial M_{P}}{\partial t}+{\left(1-f_{B}\right)}^{x}\frac{M_{R}}{\tau_{L}}-\epsilon {\left(1-f_{B}\right)}^{1-x}\frac{\partial M_{P}}{\partial t}\\ +\frac{M_{R}}{\tau_{L}}-{\left(1-f_{B}\right)}^{x}\frac{M_{R}}{\tau_{L}}+\frac{M_{R}}{\tau_{C}} \end{array}$$

Removing another set of cancelled terms

$$0=-\epsilon {\left(1-f_{B}\right)}^{1-x}\frac{\partial M_{P}}{\partial t}+\frac{M_{R}}{\tau_{L}}+\frac{M_{R}}{\tau_{C}}$$

Rearranging and solving for $M_{R}$

$$\epsilon {\left(1-f_{B}\right)}^{1-x}\frac{\partial M_{P}}{\partial t}=\frac{M_{R}}{\tau_{L}}+\frac{M_{R}}{\tau_{C}}$$

$$\frac{\epsilon {\left(1-f_{B}\right)}^{\left(1-x\right)}}{\frac{1}{\tau_{L}}+\frac{1}{\tau_{C}}}\frac{\partial M_{P}}{\partial t}=M_{R}$$ (12)

Equation 12 thus provides an analytical solution for the mass of trapped hydrogen in the subsurface ($M_{R}$) before human exploration (steady state; Fig. 3).

Anthropogenic exploration and production of hydrogen would disrupt the steady-state solution obtained in Eq. 12. This requires the incorporation of transient losses of trapped hydrogen due to resource exploitation and the counterbalancing effect of refilling of these traps due to continued hydrogen migration from deep generation. Thus, we seek to obtain a function for the change in mass of trapped hydrogen with time, $\frac{\partial M_{R}}{\partial t}$. Before any human exploration, this flux of trapped hydrogen depends only on the flux of hydrogen leaking from the traps, $\frac{\partial M_{L}}{\partial t}$, and a term describing the refilling of deep, geologically produced hydrogen denoted by $\frac{\partial M_{F}}{\partial t}$

$$\frac{\partial M_{R}}{\partial t}=\frac{\partial M_{F}}{\partial t}-\frac{\partial M_{L}}{\partial t}$$ (13)

Using the terms in fig. S1 for $\frac{\partial M_{F}}{\partial t}$, the mass balance equation for the change in trapped hydrogen with time is

$$\frac{\partial M_{R}}{\partial t}=\epsilon {\left(1-f_{B}\right)}^{\left(1-x\right)}\frac{\partial M_{P}}{\partial t}-\frac{M_{R}}{\tau_{L}}-\frac{M_{R}}{\tau_{C}}$$ (14)

Last, an additional term can be added to capture anthropogenic production, giving the master mass balance equation used for this study

$$\frac{\partial M_{R}}{\partial t}=\epsilon {\left(1-f_{B}\right)}^{\left(1-x\right)}\frac{\partial M_{P}}{\partial t}-\frac{M_{R}}{\tau }-\frac{M_{R}}{\tau_{C}}-\frac{\partial M_{D}}{\partial t}$$ (15)

This can be checked with the global mass balance equations above. In the steady state, $\frac{\partial M_{R}}{\partial t}=0$ and $\frac{\partial M_{D}}{\partial t}=0$. This reduces to

$$0=\epsilon {\left(1-f_{B}\right)}^{\left(1-x\right)}\frac{\partial M_{P}}{\partial t}-\frac{M_{R}}{\tau }-\frac{M_{R}}{\tau_{C}}$$ (16)

which is equivalent to Eq. 12.

### Numerical modeling methodology

The mass of trapped hydrogen in the subsurface through time is described by Eq. 14. This is an ordinary differential equation and was solved using a fourth-order Runge-Kutta algorithm (*25*). A MATLAB (MathWorks, Natick, MA) routine was created with the following broad steps:

1) Define input distributions for ϵ, $f_{B}$, x, τ, and $\frac{\partial M_{P}}{\partial t}$. These are shown in fig. S2 and described in the main text (Table 1).

2) Define the anthropogenic production trend, shown in Fig. 4.

3) In a parallel “for loop,” run a Monte Carlo simulation (50,000 runs) that solves Eq. 14 using the Runge-Kutta algorithm.

4) Postprocess results.

All MATLAB scripts used to calculate the model outputs are available in the Supplementary Materials.

### Estimation of input ranges

The conceptual model contains inputs that include the annual generation of natural hydrogen, the fraction of hydrogen that can be detained in traps, the residence time in reservoirs, the proportion of hydrogen lost through biotic and abiotic processes, and the rate of withdrawal associated with anthropogenic exploitation (Fig. 1). Given the extensive uncertainty associated with hydrogen generation rates, trapping efficiency, and residence times, these inputs were represented with normal distributions on a logarithmic scale (fig. S2). This produces a log-normal distribution on a linear scale, with preferential sampling focused on the low end. The loss of hydrogen and the rate of hypothetical anthropogenic production are better constrained and represented by a linear distribution (fig. S2) and analog production curve (Fig. 4), respectively. The model is assumed to be at steady state with respect to the hydrogen flux before anthropogenic withdrawals from reservoirs. Ranges of input values into the model, as derived from studies of natural hydrogen occurrences and geologic analogs, are described below and summarized in Table 1. The calculated outputs of the model are the amount of hydrogen stored in reservoirs at a given time and the flux to the atmosphere.

#### Natural hydrogen generation

The scarcity of native hydrogen associated with hydrocarbon gases has fostered a persistent perception that it does not occur on Earth (*12*). More than 30 natural processes capable of generating hydrogen have been identified, although most are thought to produce small amounts (*26*). A recent review of the occurrence of natural hydrogen on Earth estimated the annual global production from geologic environments to be 23 ± 8 Mt year^−1^ (*16*). This estimate is based on a limited number of laboratory experiments and field observations that have been extrapolated to the global scale. Several lines of reasoning support the notion that the current estimate of annual hydrogen generation in geologic settings is a minimum value. Geologic settings that are capable of generating the largest amounts of hydrogen are underexplored for gas resources, and accidental discoveries are often unreported (*27*). The earliest published estimate of global geologic hydrogen production, in 1983, was a mere 0.027 Mt year^−1^ (*28*), and every subsequent study has predicted an increased amount typically by an order of magnitude or more (*16*, *29*–*31*). Historically, subsequent observations of fluxes of hydrogen from the subsurface, which can be a proxy for generation rate in some settings, have frequently recorded larger volumes than previously detected. For example, a recent discovery in a chromite mine in Albania measured an annual flux of hydrogen more than two orders of magnitude greater than any previous observation from an ophiolite setting (0.3667 versus 0.0018 ton m^−2^ year^−1^) (*32*). Areas of microseepage of hydrogen also provide some additional evidence for the magnitude of annual hydrogen generation in local areas. For example, hydrogen flux to the atmosphere at one site in Russia was found to be ~0.25 ± 0.03 Mt year^−1^ km^−2^ (*18*) and ~1.15 ± 0.15 Mt year^−1^ km^−2^ was recorded at a site in Brazil (*19*). Estimates of hydrogen flux from these two sites alone are equivalent to ~15% of the total flux from all known volcanic and hydrothermal settings (*17*). It is important to note that translation of surface flux measurements to deep subsurface generation rates is complicated by the potential for near-surface hydrogen generation (*33*) and uncertainties in diffusion models (*34*), as well as the potential for substantial consumption of hydrogen by biotic and abiotic processes along migration pathways (*35*–*37*) (see the “Biotic and abiotic loss” section). This leads to the reasonable inference that observed fluxes of hydrogen in the near subsurface are likely reflective of much larger subsurface generation rates. Additionally, nearly all published measurements of hydrogen flux have been short term (minutes to hours). Limited time-series observations of hydrogen flux have shown that it can be highly sporadic, demonstrating that instantaneous measurements may not capture the maximum flux [see, for example, (*19*)]. Furthermore, the published values for rates of hydrogen generation in the subsurface are generally conservative minimal values (*38*).

Additionally, recent studies indicate that hydrogen generation is associated with more lithologies and under wider environmental conditions than previously recognized. For example, serpentinization-type reactions involving the reduction of water by iron-rich minerals have generally been regarded as requiring high temperatures (>~200°C) (*39*). However, there is growing evidence that these reactions can take place at much lower temperature conditions (<<200°C) [see, for example, (*40*) and references therein], which suggests that a much larger volume of rock is likely capable of generating hydrogen via serpentinization reactions than is accounted for in current estimates of annual global generation. Furthermore, other lithologies not previously accounted for in global generation estimates have recently been proposed as candidates for generation of substantial amounts of hydrogen including the reduction of water by iron-rich minerals in banded iron formations (*41*, *42*) and high–thermal maturity organic-rich rocks (*43*, *44*). Mahlstedt *et al.* (*44*) estimate that the overmature (i.e., beyond hydrocarbon generation) Patchawarra Formation in the Cooper Basin in Australia may contain a concentration of molecular hydrogen that is twice the natural gas concentration of the prolific Barnett Shale in Texas, United States.

A major uncertainty is the potential flux of hydrogen from deep crustal faults that may be conduits for fluids upwelling from the mantle. Although the upper mantle is largely oxic with H–C–O existing as CO_2_ and H_2_O in shallow regions, mantle heterogeneity and nonideal mixing provide potential for a high degree of variability in the *f*o_2_ (oxygen fugacity) of the mantle (*45*). Theoretical calculations indicate that at pressures >3 GPa (~100 km depth), CH_4_, H_2_O, and H_2_ are stable, with H_2_ constituting ~0.05 mole fraction of the fluid (*46*). The upper mantle is estimated to contain 0.04 of the Earth’s surface ocean mass of water (*47*), which could equate to ~300 × 10^6^ Mt of H_2_. Numerous experimental studies demonstrate the potential for H_2_ generation and stability under mantle conditions (*48*–*54*). Additionally, there is evidence for hydrogen-rich gas from the solar nebula to have been incorporated into Earth during early planetary accretion. Hydrodynamic escape is thought to have resulted in early loss of large amounts of nebular volatiles such as hydrogen (*55*), yet noble gas and stable isotopic geochemical data support the notion that the current endowment of hydrogen on Earth was derived from a mixture of primordial and accreted (chondritic) hydrogen (*56*–*60*). Last, theoretical models (*61*–*63*) and experimental studies (*22*, *64*–*67*) have shown that large amounts of hydrogen may be incorporated into the Earth’s core as metal hydrides or H_2_O. It has been estimated that the core could contain as much as five ocean volumes of water (*62*). The amount of H_2_ generated or stored in the mantle and core that could be transported to the crust is completely unconstrained. Noble gases are known to reach the crust from the mantle (*55*) and even the core (*68*); however, H_2_ is highly reactive and susceptible to redox conditions, so preservation throughout migration is a risk. Nonetheless, the magnitude of the reservoir of hydrogen in the deep interior of Earth is likely to be quite large and even a small fraction that escapes to the crust could constitute a substantial source for crustal accumulations.

For these reasons, we assume that the current estimate of annual hydrogen generation in geologic settings [23 ± 8 Mt year^−1^ (*16*)] is a minimum value and that the actual value may be up to three orders of magnitude larger. Consequently, we set the maximum generation rate at 25,000 Mt year^−1^. We also infer that the mean value for annual hydrogen generation is likely to be much closer to the current estimate given that the maximum generation rate likely requires a substantial contribution from a deep hydrogen source (i.e., mantle and core), which is highly uncertain. The model uses 500 and 25 Mt year^−1^ for the median and minimum input values, respectively (Table 1 and fig. S2). Sensitivity tests exploring the impact of the highly uncertain maximum generation rate value show that model results are relatively insensitive to this number, with rates of 2500, 25,000, and 250,000 Mt year^−1^ all still resulting in a median (P50) subsurface hydrogen resource estimate of ~5.6 × 10^6^ Mt.

#### Fraction trapped

Some fraction of the total volume of hydrogen moving through the subsurface will migrate into geologic traps, and the balance will escape toward the surface, which is referred to as the trapping efficiency. Trapping efficiency has been studied in petroleum systems and found to be highly variable, with amounts trapped in individual catchments ranging from 0 to 66% of the amount generated (*69*). The maximum trapping efficiency for an entire petroleum system has been estimated to be as high as ~35% (*70*, *71*); however, other authors have suggested that the maximum is more likely to be closer to ~10%, with average values of a few percent being the most common (*72*, *73*). On the basis of a comprehensive study of 16 petroleum systems from around the world, Magoon and Valin (*70*) classified petroleum systems as very efficient (>10%), moderately efficient (1 to 10%), and inefficient (<1%). Given the possibility that hydrogen trapping may be less efficient than petroleum, input values for our model ranging from 0.1 to 10% are taken to be a conservative estimate of hydrogen trapping efficiency (Table 1 and fig. S2).

#### Physical losses from reservoirs

Trapped hydrogen may escape over time due to leakage through reservoir seals, and the flux of hydrogen out of reservoirs can be accounted for by a residence time in the reservoir. Although the small size of hydrogen atoms has led to speculation that molecular hydrogen easily diffuses through most materials (*16*), there is evidence for a natural gas accumulation in Australia containing ~11% hydrogen having been preserved for millions of years (*74*). The kinetic diameter of molecular hydrogen (H_2_) is similar to that of a helium atom (*75*), and the diffusivities of these species through natural materials are similar (*76*). Low-permeability seals, such as evaporites and carbonates, allow natural helium accumulations to be trapped for long periods of time [>100 million years (Myr)] without notable diffusive leakage (*77*–*79*). Additionally, the capillary entry pressure required to force helium gas through seal rocks is similar to that of CO_2_ (*80*), suggesting that natural CO_2_ accumulations are also appropriate analogs for gas-phase hydrogen resources. CO_2_ accumulations have been shown to be in place for >100 Myr (*81*). In addition to diffusive loss, hydrogen loss may occur through advective processes. The residence time for hydrogen-filled reservoirs with advective gas loss through leaky seals was estimated to be 1 × 10^4^ years in one recent model of natural hydrogen accumulation (*82*). The model input range for the residence time associated with leakage of hydrogen trapped in reservoirs is taken to be 1 × 10^5^ years to 5 billion years (Gyr) (Table 1 and fig. S2).

#### Biotic and abiotic loss

The loss of hydrogen through biologic and abiotic processes is captured in the consumption terms of the model, which have been broken down into three components to account for the complex and potentially substantial role of consumption of hydrogen in the subsurface. Two components of the model focus on consumption that may occur while hydrogen is migrating through the subsurface, whereas the third component treats consumption that may occur while hydrogen is stored within reservoirs. The treatment of consumption of hydrogen during migration is considered for both deep or high-temperature regions (driven by abiotic consumption) versus shallow or low-temperature regions (driven by biotic consumption). The most widely recognized mechanisms for abiotic destruction of H_2_ in nature involve the catalytic hydrogenation of CO or CO_2_ at elevated temperatures (*83*), which is analogous to engineered hydrocarbon synthesis processes involving metallic iron and nickel known as Fischer-Tropsch and Sabatier synthesis, respectively (*84*). However, the prevalence of effective catalysts (e.g., Fe-Ni alloys) in natural environments has been questioned (*85*), and if they are present, the operative ranges of temperature and water-to-rock ratios are thought to be quite narrow (*39*). Furthermore, sustained catalytic reactions require a high surface area of the metal (to maximize reactive sites), low sulfur concentrations (to avoid catalyst poisoning), and low hydrogen-to-carbon ratios (to reduce coke deposition) (*86*). These conditions can be controlled in laboratory and industrial settings but are likely to be rare in natural environments (*85*). Thus, the model assumes that hydrogen consumption at greater depth (i.e., higher temperatures) is much less efficient than hydrogen consumption in shallower cooler settings where microbial processes are most effective (<120°C) (*87*). Deep hydrogen consumption during migration is estimated to constitute from 0.1 to 10% of the total hydrogen consumption (Table 1 and fig. S2).

There is a growing recognition that substantial microbial communities capable of utilizing and producing hydrogen exist in the subsurface (*35*), yet studies of the magnitude of subsurface microbial hydrogen consumption are limited and restricted to a few geologic settings. One study of the Juan de Fuca Ridge in the eastern Pacific Ocean found that microbes consume ~50 to 80% of the locally produced hydrogen (*88*), and another on the Mid-Atlantic Ridge estimated hydrogen consumption approaching approximately 90% of the production (*89*). A global model of hydrogen sources and sinks at mid-ocean ridges conservatively estimates the minimum amount of microbial consumption in these settings to be ~30% of the produced hydrogen (*90*). A case study based on laboratory incubations of soils from the São Francisco Basin in Brazil predicted a 40% reduction in hydrogen concentration in the upper 1 m of soil and noted that the observed rate was three to four orders of magnitude lower than previous studies of low-affinity hydrogen consumers (*91*). Recent work on the Samail ophiolite in Oman has observed active hydrogenotrophy capable of reducing aqueous hydrogen concentrations by six orders of magnitude over just a few hundreds of meters depth range (*92*, *93*). Paradoxically, the known hydrogen accumulation in Mali contains nearly pure hydrogen gas in a reservoir that is only a few hundred meters deep (*9*), highlighting the importance of other environmental factors (e.g., aqueous media and nutrient availability) in controlling the rate of microbial hydrogen consumption (*35*). Although not strictly consumption, the sorption of hydrogen on clay minerals is another potential mechanism for loss of hydrogen at lower temperatures (*94*). The total hydrogen consumption from combined deep (primarily abiotic) and shallow (primarily biologic) migration is assumed to range from 90 to 99.999% in the model (Table 1 and fig. S2).

Microbial consumption of fluids stored in reservoirs at low temperature (<80°C) is a well-established phenomenon in petroleum geology, wherein long-chain hydrocarbons are consumed as methane is produced (*95*). An analogous loss of hydrogen while stored in shallow, low-temperature reservoirs is likely to also occur and is represented here with a second residence time. Because biodegradation rates are poorly constrained, even in petroleum systems, the estimated rate from Larter *et al.* (*95*) of 10^−3^ to 10^−4^ kg petroleum m^−2^ year^−1^ is used, following a methodology similar to that used by Prinzhofer and Cacas-Stentz (*82*). Taking the mid-range of this estimate and using a petroleum density of 700 kg m^−3^, the mid-case residence time is calculated to be 1.4 × 10^6^ year^−1^. Although far more rapid rates for biological consumption of hydrogen have been reported in soils [as rapid as weeks (*91*)], reservoirs, traps, and especially seals of subsurface fluid accumulations are only likely in bedrock layers underlying soil. Many traps are likely to be at greater depth with temperatures >100°C, precluding a major role for microbial activity within the reservoir (*95*). Thus, the model input range for the residence time associated with in-reservoir consumption is taken to be 1 × 10^4^ years to 5 Gyr (Table 1 and fig. S2).

#### Anthropogenic hydrogen production potential

Similar to early exploration efforts for other commodities, exploration for geologic hydrogen will likely proceed slowly at first since new concepts for the geologic hydrogen system and prospect definition are still being developed (*96*). However, as this system is better understood through research, development, and prospect testing, production of geologic hydrogen will likely accelerate. Extraction of potential hydrogen gas resources is modeled on historical natural gas production and is referred to as exploration/production efficiency. US shale gas production is taken as an analog for early hydrogen development (*24*). Admittedly, there are notable differences between development of shale gas and natural hydrogen resources. In the case of shale gas, the location of the resource was well known, and successful production was dependent on the development of efficient engineering solutions to extract it. In contrast, the location of potential hydrogen accumulations is unknown, yet once located they are likely to be producible with technologies similar to those used for natural gas. Nonetheless, it can reasonably be assumed that there will be a period of low initial hydrogen production, as was experienced in shale gas development, that reflects the learning curve of the evolution of exploration strategies.

Model values for the later more mature phase of hydrogen production are based on the global natural gas production from 1973 to 2020 (*97*). Both the US shale gas and global natural gas production datasets were converted from cubic feet of natural gas to cubic feet of hydrogen and then to million metric tons of hydrogen. We used a piecewise linear curve to represent this probable production history. From 2020 to 2050, we follow the ramp-up of US shale gas (we assume that some shale gas production began as early as 1980 and then follow the available data trend from 2000 to 2023), whereas from 2050 to 2200, we follow the approximately linear increase in gas production globally (analog data from 1973 to 2020). This production curve is initiated at 2020 in the model, and the final piecewise linear curve is shown in Fig. 4.

The percent of in-place resource recoverable (i.e., recovery factor) for oil is thought to be approximately 30 to 35% (*98*) but is substantially higher for natural gas accumulations, typically ~50 to 80% (*99*). To account for the likelihood of subeconomic hydrogen accumulations (i.e., too small, too deep, and too far offshore), exploration inefficiency (i.e., inability to locate economic accumulations), and a 50 to 80% recovery factor, the maximum production amount is capped at 10% of subsurface reservoir amount.

Predicted trends for future demand and production of hydrogen provide some insight into the potential for geologic hydrogen to meet the future demand. To provide a baseline on possible demand for geologic hydrogen, a comparison was drawn from blue hydrogen. The International Energy Agency (IEA) projection of the supply of blue hydrogen over the coming decades needed to reach net-zero carbon emission goals is used as a reference case (*1*). The IEA projections for total supply of hydrogen-based fuels in 2020, 2030, and 2050 are 87, 212, and 528 Mt, respectively (*1*), and the projections for the role of blue generation in the same years are 0.7, 28, and 36% of the total. Thus, the total production of blue hydrogen is projected to be 0.63, 60, and 190 Mt (for 2020, 2030, and 2050, respectively). To set realistic amounts for a new technology such as geologic hydrogen exploration, we divided these values in half as a desirable benchmark for future demand. Comparison of the model of geologic hydrogen production with the reference case is shown in Fig. 4.

Using the model inputs specified in Table 1 and fig. S2, potential subsurface hydrogen resource estimates range from 10^3^ to 10^10^ Mt, with the most likely value of ~5.6 × 10^6^ Mt (Fig. 3). Meanwhile, the estimated future annual demand beyond 2100 may be several hundred million metric tons (Fig. 4). Although the in-place resource is expected to be sufficiently high compared to potential production, we nevertheless expect future exploration to be imperfect and therefore have constrained the model with an “exploration cap.” This serves as a limit on how efficient we might be as explorers and limited the model to extracting no more than 10% of the global in-place resource at any time. When this cap is reached, the modeled production declines, similar to expectations for oil production following “peak oil.” Figure S3 illustrates the suite of model simulations’ production curves; modeled production follows the specified production curve (Eq. 14) until the exploration cap of 10% is reached, at which point production declines to near zero. Figure S4 shows the percent of model runs that have reached the exploration cap through time. By 2100, the model predicts a probability of >94% that we will continue to meet the expected demand and will not be limited by this exploration cap. By 2200, that probability is ~75%.

The model considers the transient effect of anthropogenic production on reducing the in-place resource of geologic hydrogen. When this mass of trapped hydrogen is reduced, continued generation and trapping of hydrogen may replenish the reservoirs ($M_{F}$). We term this refilling rate as the “renewable” component of hydrogen. This annual renewable flux is illustrated in fig. S3 as the near-zero stable annual production after the exploration cap has been reached. The annual renewable flux is shown in Fig. 5 and has a P50 of 5 Mt year^−1^ with a log-normal distribution.

### Calculation of energy equivalence

The Energy Information Administration estimates global proven reserves of natural gas to be 7257 trillion cubic feet as of 1 January 2020 (*100*), which equates to 205.5 trillion m^3^. Using a density of 0.78 kg m^−3^ (*101*), this equates to 1.6 × 10^14^ kg of natural gas. The energy content of natural gas is variable, but assuming an average value of 52.2 MJ kg^−1^ from the Argonne National Laboratory GREET model (*102*), all the proven natural gas reserves of the world contain approximately 8.4 × 10^15^ J of energy. Using an energy density value for hydrogen of 141.9 MJ kg^−1^ (*102*), we can estimate that if 2% of the most probable amount of in-place geologic hydrogen (~5.6 × 10^6^ Mt) could be recovered, that would amount to ~100,000 Mt of hydrogen, which contains about 1.4 × 10^16^ J of energy, or roughly twice as much energy as is stored in all the proven natural gas reserves on Earth.

## Supplementary Materials

### This PDF file includes:

MATLAB Scripts for Model Solution Figs. S1 to S4 Table S1
