# Supplementary material for: Model predictions of global geologic hydrogen resources
Source: Sci Adv. 2024 Dec 13;10(50):eado0955. doi: 10.1126/sciadv.ado0955 (PMC11641016; doi:10.1126/sciadv.ado0955)
Supplement: Supplementary file 1 — MATLAB Scripts for Model Solution Figs. S1 to S4 Table S1 [file sciadv.ado0955_sm.pdf]

Science Advances
AAAS

Supplementary Materials for

Model predictions of global geologic hydrogen resources

Geoffrey S. Ellis and Sarah E. Gelman

Corresponding author: Geoffrey S. Ellis, gsellis@usgs.gov

Sci. Adv. **10**, eado0955 (2024)
DOI: 10.1126/sciadv.ado0955

This PDF file includes:

MATLAB Scripts for Model Solution
Figs. S1 to S4
Table S1

Matlab Scripts for Model Solution

The following scripts were written in Matlab (version 2023a) to solve the equations described in this Supplementary Materials, Derivation of Mass Balance Model Equation.

There is a main script (main_H2global_4release.m) that calls a function which solves Equation 12 via a 4th Order Runge Kutta algorithm (f_globalsupply_4release.m). This function calls a function to calculate the actual mass balances (H2massbal_4release.m), which itself calls a function to calculate trapping efficiency (H2trappeff_4release.m). These are enclosed below.

```
% Main_H2global_4release.m
clear all; close all; clc;
format long g

%% Set up the input distributions.

% Inputs to global geologic model [min mean max] for Gaussian
% distributions.
dMp = [25, 500, 25000];              % Natural/geologic production of H2 annually
[Mtons].
tau_leak_yrs = [1e5, 5e7, 5e9];     % Residence time of H2 in traps (years).
tau_cons_yrs = [1e4, 1.4e6, 5e9];    % Residence time of H2 in traps due to biotic
consumption (years).
epsilon = [0.001, 0.01, 0.1];        % Trapping efficiency (fraction).

bactH2 = [0.9, 0.95, 0.99999];       % Percent of H2 in subsurface eaten by microbes.
bactx = [0.9, 0.99, 0.999];          % Proportions bacterial consumption between
shallow and deep subsurface.
bactx_deep = 1-bactx;

%% Placeholders and settings.

Years = 2020:3000;                   % Years of calculation.
DemandStartYear = 2020;              % When we want to start exploring for H2.

Capped_percent = 10;                     % Total maximum percent of H2 natural
resource that can be recovered.

% Indices for years of interest.
DemandStartInd = find(Years==DemandStartYear);
Yr2030Ind = find(Years==2030);
Yr2050Ind = find(Years==2050);
Yr2100Ind = find(Years==2100);
Yr2150Ind = find(Years==2150);
Yr2200Ind = find(Years==2200);
Yr2300Ind = find(Years==2300);
Yr2500Ind = find(Years==2500);
Yr3000Ind = find(Years==3000);

%% Define the anthropogenic exploration/production curve and import analogues from
natural gas.

% Conversion factors for the global natural gas data to equivalent Mtons of H2.
H2_density = 0.08;     % kg/m^3
feet_2_meter = 0.3048;
kg_2_ton = 0.001102;
m3_2_cf = 0.02832;

% Placeholder vectors for anthropogenic exploration/production scenarios.
dMd = zeros(length(Years),1);            % Anthropogenic demand
(exploration/production) scenario.
dMd_bluefull = zeros(length(Years),1);   % Blue H2 projection from IEA
(extrapolated).

% Piecewise linear anthropogenic exploration/production curve to match both
```

```
% US Shale Gas and Global Natural Gas H2 equivalent mass.
dMd(DemandStartInd:Yr2050Ind) = (Years(DemandStartInd:Yr2050Ind)-
DemandStartYear)*(12/30);
dMd(Yr2050Ind+1:Yr2200Ind) = dMd(Yr2050Ind) + (Years(Yr2050Ind+1:Yr2200Ind)-
2050)*(738/150);
dMd(Yr2200Ind+1:end) = dMd(Yr2200Ind) + (Years(Yr2200Ind+1:end)-
2200)*(1000/(Years(end)-2200));

% Benchmark from Blue H2 (IEA).
dMd0_i = 0.6;                          % Mtons of produced Blue H2 in 2020.
DemandGrowth_20_30 = 5.9;              % Increase rate in 1/2 Blue H2 from 2020-
2030.
DemandGrowth_30_50 = 6.5;              % Increase rate in 1/2 Blue H2 from 2030-
2050.
dMd_bluefull(DemandStartInd:Yr2030Ind)=dMd0_i +
DemandGrowth_20_30*(Years(DemandStartInd:Yr2030Ind)-DemandStartYear);
dMd_bluefull(Yr2030Ind+1:end) = dMd_bluefull(Yr2030Ind) +
DemandGrowth_30_50*(Years(Yr2030Ind+1:end)-2030);
dMd_bluehalf = dMd_bluefull./2;
dMde = dMd_bluehalf;

%% Plot anthropogenic exploration/production curve and analogues.
figure('Color','white');
plot(Years,dMde,'k','LineWidth',2); hold on;
plot(Years,dMd,'r-','LineWidth',2);
plot([2020, 2030, 2050],[0.63, 60, 190],'sk');
legend('1/2 Blue H2 Expected',...
    'Natural Gas Analgoue',...
    'Global Natural Gas Production (analogue 1973-2020)','US Shale Gas (analogue
2000-2023)','Location','NorthWest');
xlabel('Year'); ylabel('Mtons/year'); grid on;
axis([DemandStartYear 2200 0 dMd(Yr2200Ind)+100]);
hold off;

%% Create Gaussian (~triangular) probability distributions to sample from for the
Monte Carlo simulation.

n = 50000;                             % Number of Monte Carlo simulations.
Publication = 50,000.

% Convert to log scale for:
dMp_log10 = log10(dMp);                % Geologic deep H2 generation.
tau_leak_log10 = log10(tau_leak_yrs);    % Residence time of H2 in traps.
tau_cons_log10 = log10(tau_cons_yrs); % Residence time of H2 in traps due to
consumption.
epsilon_log10 = log10(epsilon);        % Trapping efficiency.
bactx_deep_log10 = log10(bactx_deep);    % Deep vs. Shallow Consumption factor.

% Set up the standard deviations for input parameters (assumes Gaussian
% distributions).
dMp_log10_std = (dMp_log10(3)-dMp_log10(1))/6;
tau_leak_log10_std = (tau_leak_log10(3)-tau_leak_log10(1))/6;
tau_cons_log10_std = (tau_cons_log10(3)-tau_cons_log10(1))/6;
epsilon_log10_std = (epsilon_log10(3)-epsilon_log10(1))/6;
bactH2_std = (bactH2(3)-bactH2(1))/6;
```

```
bactx_deep_log10_std = (bactx_deep_log10(3)-bactx_deep_log10(1))/6;

% dMp distribution (Deep geologic generation).
dMp_log10_dist = dMp_log10_std*randn(n,1)+dMp_log10(2);
[~,dMp_log10_edges] = histcounts(dMp_log10_dist,30);

% tau distribution (Residence time in traps).
tau_leak_log10_dist = tau_leak_log10_std*randn(n,1)+tau_leak_log10(2);
[~,tau_leak_log10_edges] = histcounts(tau_leak_log10_dist,30);

% tau consumption distribution (Residence time in traps due to consumption).
tau_cons_log10_dist = tau_cons_log10_std*randn(n,1)+tau_cons_log10(2);
[~,tau_cons_log10_edges] = histcounts(tau_cons_log10_dist,30);

% epsilon distrubution (Trapping efficiency).
epsilon_log10_dist = epsilon_log10_std*randn(n,1)+epsilon_log10(2);
[~,epsilon_log10_edges] = histcounts(epsilon_log10_dist,30);

% bacterial consumption distribution.
bactH2_dist = bactH2_std*randn(n,1)+bactH2(2);
bactH2_dist = min(bactH2_dist,ones(n,1));  % Ensure we do not exceed 1.
[~,bactH2_edges] = histcounts(bactH2_dist,30);

% bacterial consumption distribution SHALLOW VS DEEP.
bactx_deep_log10_dist = bactx_deep_log10_std*randn(n,1)+bactx_deep_log10(2);
[~,bactx_deep_log10_edges] = histcounts(bactx_deep_log10_dist,30);


% Plot these sampling distributions.
figure('Color','white');
subplot(2,3,1);
histogram(10.^(dMp_log10_dist),10.^dMp_log10_edges); title('Generation Flux
Distribution (dM_P/dt)'); hold on;
plot(dMp,0,'ko','MarkerFaceColor','k'); grid on;
ax = gca; ax.XScale = 'log';
xlabel('Geologic Generative Flux (Mtons/yr)'); ylabel('Number of Runs'); hold off;

subplot(2,3,2);
histogram(10.^(tau_leak_log10_dist),10.^tau_leak_log10_edges); hold on;
plot(tau_leak_yrs,0,'ko','MarkerFaceColor','k'); grid on;
set(gca, 'xscale','log'); title('Leak Residence Time (\tau_L) Distribution');
xlabel('H_2 Leak Residence Time (Years)'); ylabel('Number of Runs'); hold off;

subplot(2,3,3);
histogram(10.^(tau_cons_log10_dist),10.^tau_cons_log10_edges); hold on;
plot(tau_cons_yrs,0,'ko','MarkerFaceColor','k'); grid on;
set(gca, 'xscale','log'); title('Consumption Residence Time (\tau_C) Distribution');
xlabel('H_2 Consumption Residence Time (Years)'); ylabel('Number of Runs'); hold off;

subplot(2,3,4);
histogram(10.^(epsilon_log10_dist),10.^epsilon_log10_edges); hold on;
plot(epsilon,0,'ko','MarkerFaceColor','k');grid on;
set(gca, 'xscale','log'); title('Trapping Efficiency (\epsilon) Distribution');
xlabel('Proportion of Trapped H_2 (Fraction)'); ylabel('Number of Runs'); hold off;
```

```
subplot(2,3,5);
histogram(bactH2_dist,bactH2_edges); hold on;
plot(bactH2,0,'ko','MarkerFaceColor','k');grid on;
title('Consumption (f_B) Distribution');
xlabel('Biotic/Abiotic Consumption (Fraction)'); ylabel('Number of Runs'); hold off;

subplot(2,3,6);
histogram(10.^(bactx_deep_log10_dist),10.^bactx_deep_log10_edges); title('Shallow vs.
Deep Consumption (x) Distribution'); hold on;
plot(bactx_deep,0,'ko','MarkerFaceColor','k'); grid on;
ax = gca; ax.XScale = 'log';
xlabel('Proportion of Shallow Consumption (Fraction)'); ylabel('Number of Runs');
hold off;

bactx_dist = 1-(10.^(bactx_deep_log10_dist));

%% Call on the H2 supply calculation and store results.

% Set up results placeholders.
yrdemmet = zeros(n,1);
yrdemNOTmet = zeros(n,1);
demmet2100 = zeros(n,1);
demandmet = zeros(n,length(Years));
renewflux = zeros(n,length(Years));
surfflux = zeros(n,length(Years));
dMdnotmet = zeros(n,1);
MR0s = zeros(n,1);

% Run the Monte Carlo simulation. Call a function that solves Equation 12
% in the Supplementary Materials.
for i = 1:n
    [dnm,dm2100,dmm,maxdMd,MR0,dmms,dMdnm,trappedflux,sflux] =
f_globalsupply_4release(10^(tau_leak_log10_dist(i)),10^(tau_cons_log10_dist(i)),...
        10^(epsilon_log10_dist(i)), 10^(dMp_log10_dist(i)), dMd, Years,
bactH2_dist(i), ...
        bactx_dist(i), Capped_percent, DemandStartYear);

    % store these results.
    yrdemmet(i) = dmms;
    yrdemNOTmet(i) = dnm;
    demmet2100(i) = dm2100;
    demandmet(i,:) = dmm';
    renewflux(i,:) = trappedflux';
    surfflux(i,:) = sflux';
    dMdnotmet(i) = dMdnm;
    MR0s(i) = MR0;

end

%% P50 and mean calculations.
% Total steady state global endowment (reservoired H2 prior to human
% production).
sorted_MR0s = sort(MR0s);
P50_MR0 = sorted_MR0s(round(0.5*n))
mean_MR0 = mean(MR0s,'omitnan')
```

```
disp('Probability of demand being met until 2100 (%):');
mean(demmet2100)*100

% Surface flux.
sorted_surfflux = sort(surfflux(:,2));
P50_surfflux = sorted_surfflux(round(0.5*n))
mean_surfflux = mean(surfflux(:,2),'omitnan')

%% Correlation coefficients.
cc_dMp = corrcoef(dMp_log10_dist,yrdemNOTmet);
cc_tau_leak = corrcoef(tau_leak_log10_dist,yrdemNOTmet);
cc_tau_cons = corrcoef(tau_cons_log10_dist,yrdemNOTmet);
cc_epsilon = corrcoef(epsilon_log10_dist,yrdemNOTmet);
cc_bactH2 = corrcoef(bactH2_dist,yrdemNOTmet);
cc_bactx = corrcoef(bactx_dist,yrdemNOTmet);

disp(strcat('Surface flux correlation coefficient: ',num2str(cc_dMp(1,2))));
disp(strcat('H2 residence time (leak) correlation coefficient:
',num2str(cc_tau_leak(1,2))));
disp(strcat('H2 residence time (consumption) correlation coefficient:
',num2str(cc_tau_cons(1,2))));
disp(strcat('Trapping efficiency correlation coefficient:
',num2str(cc_epsilon(1,2))));
disp(strcat('Bacterial consumption correlation coefficient:
',num2str(cc_bactH2(1,2))));
disp(strcat('Bacterial consumption shallow v deep correlation coeff:
',num2str(cc_bactx(1,2))));

%% Plot Annual Production.
figure('Color','white');
plot(Years, dMde, 'k', 'LineWidth', 2); hold on;
for i = 1:10:n  %(for speed, only plot every 10th run)
    plot(Years,demandmet(i,:),'k','LineWidth',0.5); hold on;
end
grid on; xlabel('Year'); ylabel('Mtons/year'); legend('Demand Desired (= 1/2 Blue
H2)');
axis([DemandStartYear 2200 0 dMd(Yr2200Ind)]);
hold off;

%% Plot Global Endowment
bins = 10.^(0.1:0.1:12);
figure('Color','white');
histogram(MR0s,bins);
set(gca, 'XScale','log'); grid on;
title('Steady-State (Pre-Demand) Global H2 in Reservoirs (Mtons)');

%% "Renewable" H2 - the refilling H2 through time.

% Calculate the P10, P50, P90 of this distribution.
sorted_renewflux = sort(renewflux(:,2));
disp('Renewable statistics:');
P90_rf = sorted_renewflux(round(0.1*n))
P50_rf = sorted_renewflux(round(0.5*n))
P10_rf = sorted_renewflux(round(0.9*n))
```

```
figure('Color','white','Position',[100 100 1000 400]);
hold on; histogram(renewflux(:,2),[0:2:150]);
plot(P90_rf*ones(2,1),[0; 4000],'k-.');
plot(P50_rf*ones(2,1),[0; 4000],'k-.');
plot(P10_rf*ones(2,1),[0; 4000],'k-.');
plot(mean(sorted_renewflux)*ones(2,1),[0; 1000],'k');
title('Renewable H2 (Mtons/yr)');
axis([0 100 0 14000]); xlabel('Steady-state Refilling H2 (Mtons/yr)'); ylabel('Number
of Runs');
text(80,300,strcat('n = ',num2str(n)));
hold off;

%% Calculate the percent of runs through time that are just on the demand curve (thus
we haven't hit any geologic constraint on H2 production).

perc_met_demand = zeros(length(Years),1);
for i = 1:length(Years)
    ct = length(find(demandmet(:,i)==dMd(i)));
    perc_met_demand(i) = ct*100/n;
end

figure('Color','white');
plot(Years,perc_met_demand,'k','LineWidth',2);
xlabel('Time (years)'); ylabel('% Runs Meeting Expected Production'); grid on;
axis([2020 2200 0 100]);

%% Check the surface fluxes.
figure('Color','white');
subplot(1,2,1);
histogram(10.^(dMp_log10_dist),10.^dMp_log10_edges); title('Geologic Flux
Distribution (dM_P/dt)'); hold on;
plot(dMp,0,'ko','MarkerFaceColor','k'); grid on;
ax = gca; ax.XScale = 'log';
ylabel('Number of Runs'); xlabel('Geologic Generative Flux (Mt/year)'); hold off;

[~,sflux_log10_edges] = histcounts(log10(surfflux),30);
subplot(1,2,2);
histogram(surfflux(:,2),10.^sflux_log10_edges); title('Surface Flux Distribution
(dM_S/dt)'); hold on;
grid on;
ax = gca; ax.XScale = 'log';
ylabel('Number of Runs'); xlabel('Surface Flux (Mt/year)'); hold off;
```

```
% Solves the ODE for Supplementary Material Equation 14 through time.

function
[yrdemNOTmet,demmet2100,demandmet,dMd,MR0,yrdemMET_start,yrdMdNOTmet,trappedflux,surf
flux] = f_globalsupply_4release(tau_l_yrs,tau_c_yrs,tp,dMp_o,dMd,...
    Years,bactH2,bactx,Capped_percent,DemandStartYear)

% tau_l_yrs ---------------------- Residence time of hydrogen in a trap due to
leakage.
% tau_c_yrs ---------------------- Residence time of hydrogen in a trap due to
consumption.
% tp ---------------------------- Trapping efficiency (fraction).
% dMp --------------------------- Geologic production of H2 annually (Mtons/year).
% dMd --------------------------- Demand of white H2 annually (Mtons/year).
% bactH2 ------------------------ Percent of H2 in subsurface eaten by microbes
(must be compensated to get surface flux estimate).
% bdeep ------------------------- Proportional split of shallow vs. deep bacterial
consumption.
% dMde -------------------------- The demand needed to match projected growth of
electrolysis.

% Factors for trapping efficiency
tpmax = tp;                           % Percent of H2 trapped when reservoirs are
empty.
tpmin = tp;                           % Percent of H2 trapped in steady state
(reservoirs only losing via diffusion).

dMp = dMp_o;

%MR0 = tau_l_yrs*dMp*tpmin*((1-bactH2)^(1-bactx));             % Reservoir capacity
of H2 stored in surbsurface in steady state (Mt).
MR0 = (dMp*tpmin*((1-bactH2)^(1-bactx)))/((1/tau_l_yrs)+(1/tau_c_yrs));

%% Now we need to solve our ODE with Runge Kutta
% Set up solution vector for reservoired H2.

MR = zeros(length(Years),1);
MR(1) = MR0;
surfflux = zeros(length(Years),1);
diffflux = zeros(length(Years),1);
bactflux = zeros(length(Years),1);
trappedflux = zeros(length(Years),1);
demandmet = zeros(length(Years),1);

h = 1;   % timestep in years.

% Set up a for-loop to increase each year and calculate.
for i = 2:length(Years)

    MRtemp = MR(i-1);
    dMdtemp = dMd(i-1);
    k1 =
H2massbal_4release(MRtemp,tau_l_yrs,tau_c_yrs,dMp,tpmax,tpmin,bactH2,bactx,MR0,dMdtem
p,Capped_percent);
```

```
    MRtemp = MR(i-1) + (h*(k1/2));
    dMdtemp = dMd(i-1) + (0.5*(dMd(i-1)+dMd(i)));
    k2 =
H2massbal_4release(MRtemp,tau_l_yrs,tau_c_yrs,dMp,tpmax,tpmin,bactH2,bactx,MR0,dMdtem
p,Capped_percent);

    MRtemp = MR(i-1) + (h*(k2/2));
    dMdtemp = dMd(i-1) + (0.5*(dMd(i-1)+dMd(i)));
    k3 =
H2massbal_4release(MRtemp,tau_l_yrs,tau_c_yrs,dMp,tpmax,tpmin,bactH2,bactx,MR0,dMdtem
p,Capped_percent);

    MRtemp = MR(i-1) + (h*k3);
    dMdtemp = dMd(i);
    k4 =
H2massbal_4release(MRtemp,tau_l_yrs,tau_c_yrs,dMp,tpmax,tpmin,bactH2,bactx,MR0,dMdtem
p,Capped_percent);

    MR(i) = MR(i-1) + ((1/6)*h*(k1+(2*k2)+(2*k3)+k4));

    % Calculate the expected surface flux.
    [dMRdt,t1,t2,t3,tp] =
H2massbal_4release(MR(i),tau_l_yrs,tau_c_yrs,dMp,tpmax,tpmin,bactH2,bactx,MR0,dMd(i),
Capped_percent);

    diffflux(i) = -t1*((1-bactH2)^bactx);
    surfflux(i) = diffflux(i) + (dMp*(1-(tp/100))*(1-bactH2));
    bactflux(i) = ((tp/100)*(1-((1-bactH2)^(1-bactx)))*dMp) - (1-((1-
bactH2)^bactx))*t1 + ((1-(tp/100))*bactH2*dMp);
    trappedflux(i) = t2;
    demandmet(i) = -t3;

end

%% Gather up pertinent results.

% We want to know:
%   - Years when demand is being met.
yrdemMET = Years(find(demandmet>=dMd));   % all the years (including before demand)
in this condition.

%   - Year when demand starts to be met.
yrdemMET_start = yrdemMET(find(yrdemMET>DemandStartYear,1,'first'));

if isempty(yrdemMET_start)                % Catch the runs where we never meet
demand...
    yrdemMET_start = DemandStartYear;
end

%   - Year when demand is no longer being met.
yrdemNOTmet = yrdemMET(end)+1;

if yrdemNOTmet == DemandStartYear           % Catch the runs where we never meet
demand...
    yrdemNOTmet = DemandStartYear;
```

```
end

%   - Did we meet demand in 2100? 0 = no, 1 = yes.
yr2100 = find(Years==2100);
if demandmet(yr2100)>=dMd(yr2100)
    demmet2100 = 1;
else
    demmet2100 = 0;
end

%   - Year when we no longer are on the demand curve.
yrdMdNOTmet = Years(find(demandmet<dMd,1,'first'));
if isempty(yrdMdNOTmet) == 1
    yrdMdNOTmet = NaN;
end
```

```
% Function for Hydrogen Conservation of Mass

function [sol,t1,t3,t4,tp] =
H2massbal_4release(MR,tau_l,tau_c,MP,tpmax,tpmin,b,bx,MR0,MD,CP)

% MR            Mass of H2 currently in subsurface reservoirs (MTons)
% tau_l         Residence time for H2 via leaking (years)
% tau_c         Residence time for H2 via consumption (years)
% MP            Mass of H2 produced geologically/naturally each year (MTons)
% tpmax         Trapping efficiency of H2 produced geologically into reservoirs (when
reservoirs empty)
% tpmin         Trapping efficiency of H2 produced geologically into reservoirs (when
reservoirs full)
% b             Fraction of H2 that is consumed biologically in subsurface
% bx            Proportion controlling shallow vs. deep biologic consumption
% MR0           Mass of H2 stored geologically at steady state (before demand)
% MD            Mass of H2 that would be explored for to meet demand (MTons)
% CP            Capped Percent - max percentage of stored amount that can be
produced/demand

% term for mass lost via leaking (residence time)
t1 = -MR/tau_l;

% term for mass lost via consumption (residence time)
t2 = -MR/tau_c;

% term for trapped amount of H2 that's produced geologically
tp = H2trapeff_4release(MR,MR0,tpmax,tpmin);
t3 = MP*((tp/100)*((1-b)^(1-bx)));

% term for production to meet human demand
% Need to make sure if there's not enough left, don't produce it!
rescalc = MR+t1+t2+t3;                      % Amount currently in reservoirs globally
resleft = rescalc - MD;                     % Amount that would be left after desired
demand
maxresdem = MR0*CP/100;                     % Total amount that can be produced to
meet demand (exploration limit)
minresdem = MR0*(100-CP)/100;               % Total amount that must be left in the
ground (exploration limit)
if resleft <= minresdem                     % If amount left after demand < total
amount that must be left in the ground (exploration limit)
    t4 = -(rescalc-minresdem);              % Demand equals amount currently in
reservoirs - amount that must remain in the ground
else
    t4 = -MD;
end

sol = t1 +t2 + t3 + t4;
```

```
% Function to create a trend for trapping efficiency of H2.

function tp = H2trapeff_4release(MR,MR0,tpmax,tpmin)

tp = 100*(((-tpmax+tpmin)*MR./MR0)+tpmax);
```

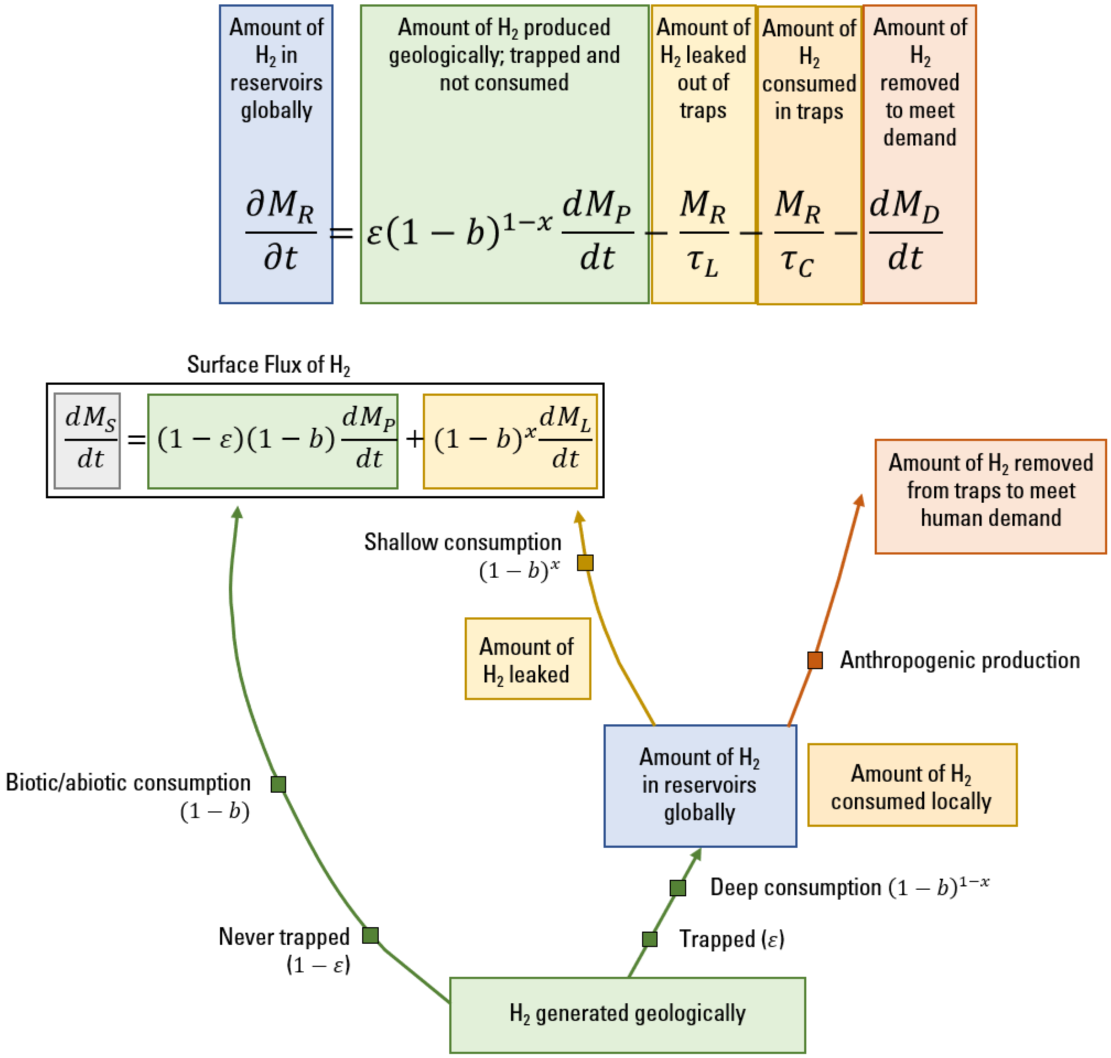

Fig. S1.

Mass balance conceptual box model utilized for the derivation of principle mass balance equations. All symbols are as defined in Table S1.

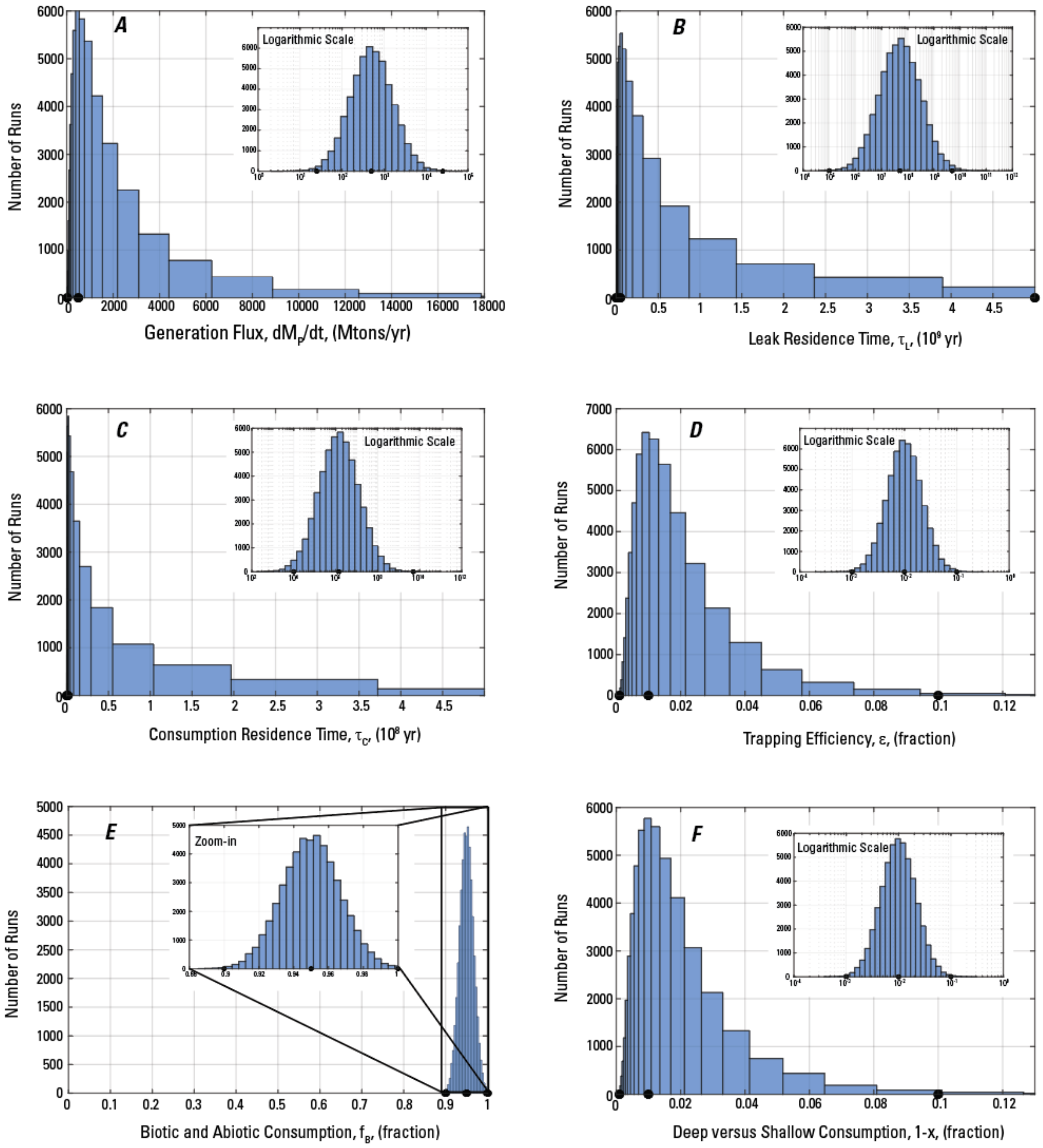

Fig. S2.

Summary of model input sampling for the Monte Carlo simulation. Black circles along the x-axis indicate low, high, and mid values for each parameter, as shown in Table 1. *A*) Generation flux on a linear scale, with inset showing sampling distribution on a logarithmic scale. *B*) Residence time associated with hydrogen leakage from traps on a linear scale, with inset showing sampling distribution on a logarithmic scale. *C*) Residence time associated with consumption within reservoirs on a linear scale, with inset showing sampling distribution on a logarithmic scale. *D*) Trapping efficiency on a linear scale, with inset showing sampling distribution on a logarithmic scale. *E*) Portion of hydrogen consumed during subsurface migration either biotically or

abiotically, with the inset showing the sampling distribution zoomed in to better illustrate distribution. *F*) Proportion of consumption occurring at deep versus shallow depths on a linear scale, with the inset showing sampling distribution on a logarithmic scale. Note that all model variables, except for f_B, are represented by log-normal distributions to accommodate the wide uncertainty in their input ranges (orders of magnitude).

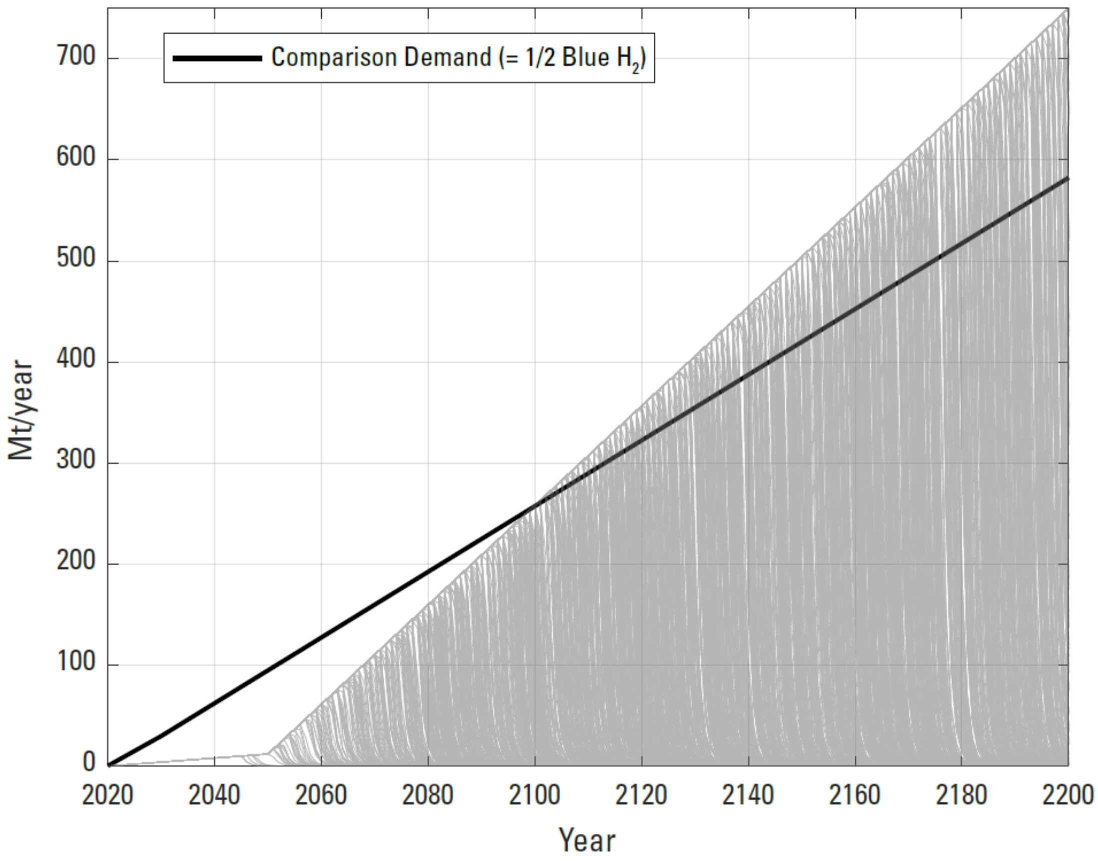

Fig. S3.

Model results for annual production. Monte Carlo simulation results are shown in thin grey, and generally follow the modeled annual production trend shown in Figure 2. However, upon reaching extraction of 10% of the in-place resource (Figure 3), production declines considerably, shown as a decline to near-zero annual production values. For comparison, a benchmark demand curve is shown for half of the IEA projection of blue hydrogen (solid black line). Note that although many simulations have this exploration cap decline as shown, this is only at most 25% of the 50,000 runs comprising the Monte Carlo simulation (Figure S4). The other 75% of the runs never reach this exploration cap and continue to follow the modeled production curve from Figure 2 but are overlain in the above figure.

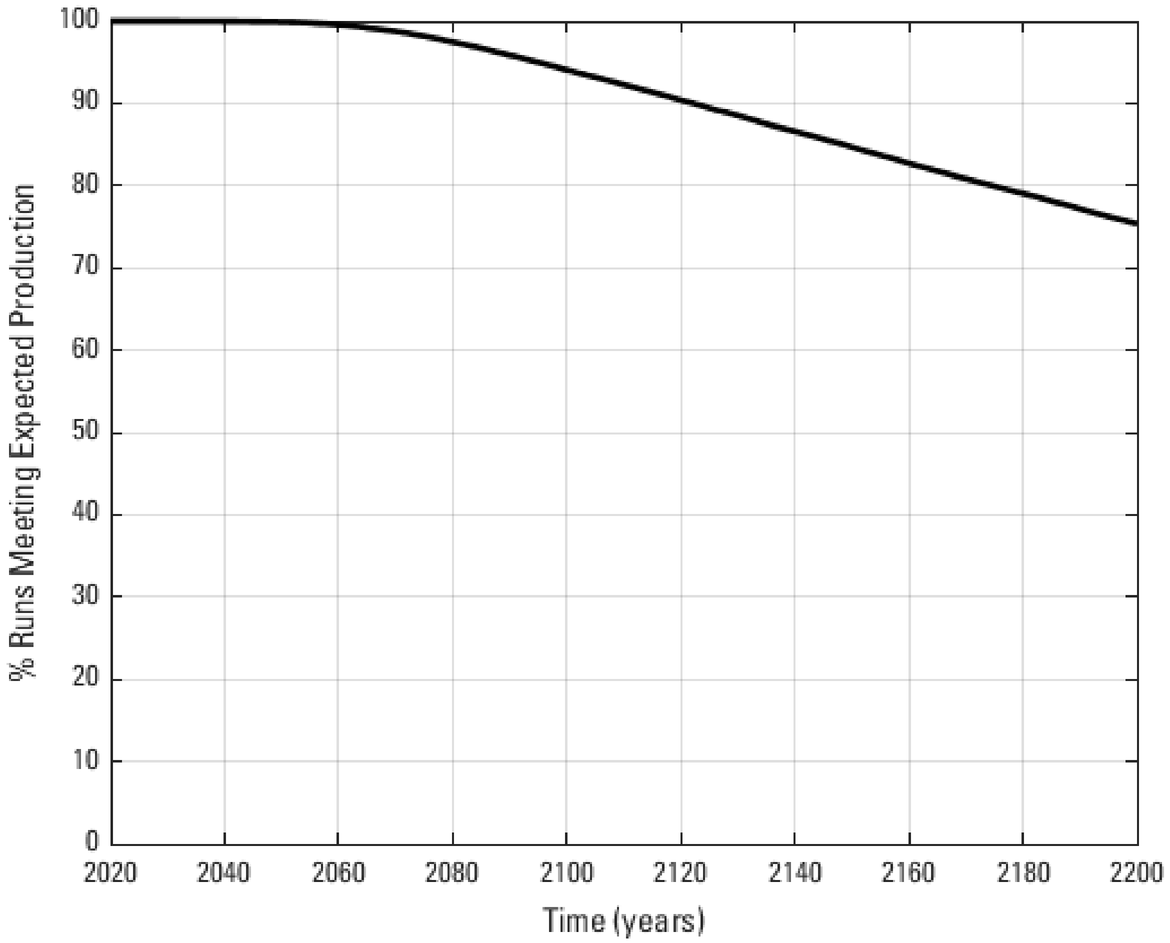

Fig. S4.

Percentage of model simulations that meet expected production (‘modeled anthropogenic production’ in Figure 2) and have not reached the 10% exploration cap imposed on the model.

Table S1.
Nomenclature utilized for derivation of mass balance equation.

Parameter	Symbol
Mass of H_2, deep geologic production	M_P
Mass of H_2, released at Earth surface	M_S
Mass of H_2, biotic/abiotic consumption	M_B
Mass of H_2, leaked from reservoirs	M_L
Mass of H_2, trapped in reservoirs	M_R
Mass of H_2, migrating into traps/reservoirs	M_F
Mass of H_2, anthropogenically produced	M_D
Time	t
Fraction of H_2 that is consumed (biotic/abiotic)	f_B
Proportion of biotic/abiotic consumption occurring in shallow subsurface	x
Trapping efficiency (fraction of H_2 trapped)	ϵ
Half-life of H_2 in reservoirs associated with physical leakage	λ_L
Half-life of H_2 in reservoirs associated with consumption	λ_C
Residence time of H_2 in reservoirs associated with physical leakage	τ_L
Residence time of H_2 in reservoirs associated with consumption	τ_C
Portion of surface H_2 flux that was never trapped in a reservoir	$\left(\frac{\partial M_S}{\partial t}\right)_{NT}$
Portion of surface H_2 flux that was trapped and leaked from reservoirs	$\left(\frac{\partial M_S}{\partial t}\right)_{L}$
Portion of H_2 consumed during migration that was never trapped	$\left(\frac{\partial M_B}{\partial t}\right)_{NT}$
Portion of H_2 consumed during migration at depth, prior to trapping	$\left(\frac{\partial M_B}{\partial t}\right)_{PTD}$
Portion of H_2 consumed during shallow migration, after leaking	$\left(\frac{\partial M_B}{\partial t}\right)_{PTS}$
Portion of H_2 consumed locally within reservoirs	$\left(\frac{\partial M_B}{\partial t}\right)_{R}$
